# The health literacy questionnaire among the aged in Changsha, China: confirmatory factor analysis

**DOI:** 10.1186/s12889-019-7563-x

**Published:** 2019-09-04

**Authors:** Yiwei Huang, Tingting Ruan, Qiaoyun Yi, Tingting Wang, Zhihua Guo

**Affiliations:** Xiangya School of Nursing, Central South University, Changsha, Hunan People’s Republic of China

**Keywords:** Health literacy, Questionnaire, HLQ, Bayesian structural equation modeling, BSEM, Psychometric

## Abstract

**Background:**

Health literacy is defined as the cognitive and social skills that determine the motivation and ability of individuals to gain access to, understand and use information in ways that promote and maintain good health. A Health Literacy Questionnaire (HLQ) is a toolkit with good reliability and validity. Accordingly, this study administered HLQ among older adults in China to examine its factor structure, reliability, homogeneity, and discriminant validity for use in understanding better the health literacy of older adults and determining corresponding measures.

**Methods:**

Psychometric properties were examined based on the data collected via face-to-face interviews (*N* = 343). Tests included the difficulty level, composite scale reliability, confirmatory factor analysis (CFA), and Bayesian structural equation modeling (BSEM).

**Results:**

The easiest scale to obtain a high score was “Social support for health” and the hardest, “Navigating the health care system” and “Appraisal of health information.” Two one-factor models fitted well with no correlated residuals allowed. After model modification, the CFA fit statistics of the other seven scales were good. All HLQ scales were found to be homogenous, with a composite reliability ranging from 0.74 to 0.85. The nine-factor Bayesian structural equation model fitted the data well (Posterior-Predictive-P value = 0.670; 95% Confidence Interval for the difference between the observed and replicated Chi-square values = − 163.320, 102.750).

**Conclusions:**

The Chinese version of the HLQ has strong construct and content validity and high composite reliability when applied to older adults in Changsha City, China. Therefore, the nine-scale HLQ can now be administered to Chinese older adults, thereby providing a powerful approach to understanding the multidimensional area of health literacy.

## Background

The World Health Organization defined health literacy as “the cognitive and social skills which determine the motivation and ability of individuals to gain access to, understand and use information in ways which promote and maintain good health” [1]. The focus on health literacy has intensified over the last two decades, especially in the areas of health systems improvement, public health, and health policy [2, 3]. In both developing and developed countries, health literacy is a key consideration for promoting health and improving the quality of health services, owing to the complexity of modern health care and the promotion of health messages [4–8].

Health literacy is important in public health and health care, playing a crucial role in the management of chronic diseases [4]. It can affect health behaviors and health outcomes directly or indirectly and be used as an independent factor in the excessive expenditure of medical expenses [9, 10]. A study pointed out that when people manage their own health, including the use of various health technologies, having a wide range of health literacy competencies becomes a necessity [11]. Research has shown that most adults will face a situation where their health literacy is inadequate in relation to the complexity of the health issues they face [2].

The older adults population is a vulnerable group with regard to health literacy. Research has indicated that functional health literacy is negatively associated with age [12]. According to an adult health literacy survey conducted in the United States, adults in the oldest age group (65 and older) had a lower average health literacy than those in the younger age groups [13]. Similarly, China’s first survey on health literacy showed a low health literacy level among older adults, with only 3.81% of people over 65 years old showing adequate health literacy skills [14]. Limited health literacy causes older adults to have insufficient understanding of chronic diseases, poor self-management of diseases, a low utilization rate of medical resources, and poor compliance with drug treatment. Low health literacy tends to cause stigma among older adults and limit their use of health care services. The implications of older adults being not treated appropriately include their worse general health conditions, their poor health care access, higher admission rates, and higher emergency rates, ultimately leading to an increase in personal and national health expenditure [10, 15–17].

Given this context, carrying out interventions for the older adults population is important to improve their health literacy levels and quality of life. Toward this end, we need to understand the status of their health literacy. However, we need to know that older adults belong to a special category and require appropriate special health literacy assessment tools, which must reflect accurately their situation and be easy to use. Current measures of health literacy focus on a limited range of health-related literacy and numeracy skills [18]. However, it has been demonstrated that health literacy is a multidimensional concept that contains a variety of cognitive, affective, social, and personal skills and attributes [19, 20]. The study of China’s health literacy started relatively late, whereas research on assessment tools lags behind. Currently, the most used health literacy assessment tool is the health literacy questionnaire for Chinese residents. The questionnaire has several items and takes a long time to be completed. The content of the questionnaire includes, among others, infant feeding and HIV prevention, which cannot reflect clearly the characteristics of older adults. There is no mature development in the dimension of critical literacy. Although this tool is commonly used for the evaluation of older adults, some items do not represent their characteristics [21].

Recent developments in the measurement of health literacy have increased its capacity to assess the needs of older adults across a more extensive dimension of health literacy. The Health Literacy Questionnaire (HLQ) was developed in Australia in 2012 using a “validity driven” approach. It was initially tested in diverse individuals in Australian communities, covering nine conceptually distinct areas of health literacy, to assess the needs and challenges of a wide range of people and organizations. It has been shown to have strong construct validity, reliability, and high acceptability to Public Health clients and clinicians [22]. The HLQ has been widely translated and applied in research, evaluation, and monitoring [11]. Daniel et al. used the HLQ to assess the health literacy of older adults with diabetes as a guide to designing health promotion programs [23]. It is envisaged to use the Chinese version of HLQ to measure the strengths and limitations of people in accessing, understanding, and using health information and health services, and determine the health literacy status of Chinese older adults. The data to be derived from this tool can be used to justify, endorse, or exclude treatments, interventions, and policies. Such responsibility requires a measurement tool and its data to be valid for older adults. It is expected that the HLQ will be a suitable tool in China. However, it will be necessary to undertake rigorous studies to confirm its applicability in each setting [24, 25].

## Methods

### Setting

A cross-sectional survey was carried out from March to May 2018 in the six districts of Changsha City (Yuelu, Tianxin, Kaifu, Furong, Yuhua, and Wangcheng). One or two communities were selected to conduct random sampling surveys of older adults within the scope of each community. The Chinese version of HLQ was used to describe the health literacy profile of and collect general information on older adults in Changsha. Data were collected by researchers through face-to-face interviews. The researchers helped the participants who could not fill out the questionnaire by themselves.

### Participants

Participants included in the study were: ① aged 60 years and above; ② residents of Changsha City or having lived in Changsha City for more than six months; ③ voluntary participation. Exclusion criteria were: ① those with mental illness or confusion; ② those with a major illness that makes them unable to cooperate; and ③ other investigators who have been involved in a similar investigation.

### HLQ

The questionnaire is divided into nine scales and has 44 items. Each dimension can be used as an assessment tool alone. The sum of the scores is the total score of health literacy. The health literacy is proportional to the score. The nine scales are:

1. Feeling understood and supported by health care providers
2. Having sufficient information to manage my health
3. Actively managing my health
4. Social support for health
5. Appraisal of health information
6. Ability to actively engage with health care providers
7. Navigating the health care system
8. Ability to find good health information
9. Understand health information well enough to know what to do

### Statistical analysis

Analyses were conducted using IBM SPSS Statistics version 22, Amos version 22.0 and Mplus version 7.4. SPSS was used to provide difficulty estimates within and across the nine scales. Some descriptive statistics were generated for every item, which can determine the range of answers as illustrated by providing difficulty estimates over and across nine levels. As the scales had two types of response options, the difficulty level of each item was calculated through two different methods. The first method, which was applicable to the scales (1–5) with agree/disagree options, was calculated as the ratio of disagree and strongly disagree responses to agree or strongly agree responses. The second method, which was applied to the other scales (6–9), was calculated as the ratio of cannot do, very difficult, or quite difficult responses to quite easy and very easy responses ([11], p., 3).

Validity is the degree to which the measured results reflect what is examined. In this study, the Short Form-12 (SF-12) Quality of Life Scale was used as the validity criterion. SPSS was used to calculate the Spearman rank correlation coefficient to observe the relationship between the HLQ questionnaire and calibration.

Reliability is an indicator to measure the stability and accuracy. The most commonly used indicator for evaluating test reliability is Cronbach’s α. As the α coefficient is often biased in population reliability estimates, it is more reliable to use a confirmatory factor analysis (CFA) to calculate the composite reliability [26]. For this, we used nine one-factor CFA models fitted to the data to confirm the scales. Composite reliability was computed by Amos with robust maximum likelihood estimation [27]. To evaluate the fitness of these models, fit indices “unstandardized and standardized” factor loadings, estimation of variance of measured variables explained by the latent variable (R^2^), Root Mean Square Error of Approximation (RMSEA), Tucker-Lewis Index (TLI), and the Comparative Fit Index (CFI) were applied. A value of 0.05 was interpreted as close fit for RMSEA and 0.08 as acceptable fit. For both TLI and CFI, a cut-off value of 0.95 was applied [28–32].

It has been argued that the traditional CFA using maximum likelihood estimation testing applies unnecessarily rigorous models to verify theory hypothesis, which often leads to rejection of the model [33]. For this study, data using the Mplus code was used to investigate whether the discriminant validity fits a specific full nine-factor Bayesian structural equation modeling (BSEM) with no correlated residuals or cross-loadings [34–36]. With the use of small variance priors, BSEM makes it available for models to adapt to flexibility to evaluate minor variations in the rigorous zero-constraints of residual correlations and cross-loadings in a typical multi-factor CFA model [35]. It is thus possible to achieve good model fitting and subsequent unbiased estimation of model parameters.

The BSEM uses a different method from the commonly used ones to assess model fit. After calculating the chi-square likelihood data, a “post-test prediction-P value” (PPP value) was generated to evaluate the model fit. In the well-fitted model, the PPP value was close to 0.5; a lower value or a value close to 0.0 indicates poor fit.

## Results

### Reliability and homogeneity of the Chinese HLQ scales

As shown in Table 1, the first five scales, compared with the last four scales, are relatively difficult at the item level. The easiest scale to obtain a high score was “4. Social support for health,” with an average item difficulty of 0.15. The hardest scales to obtain a high score were “7. Navigating the health care system” (0.56) and “8. Ability to find good health information” (0.53). The two easiest items were under “4. Social support for health”: “4.4 I have at least one person who can come to medical appointments with me” (0.11) and “4.5 I have strong support from family or friends” (0.11). The hardest item was found in scale “7. Navigating the health care system”: “7.5 Find out what health care services you are entitled to” (0.70). Scale “5. Appraisal of health information” had the smallest difficulty range (hardest: 0.40, easiest: 0.34, range: 0.06), whereas scale “7. Navigating the health care system” (hardest: 0.70, easiest: 0.46, range: 0.24) and “9. Understanding health information well enough to know what to do” (hardest: 0.55, easiest: 0.31, range: 0.24) had the widest difficulty range (Table 1).

**Table 1:** Data quality of the translated Health Literacy Questionnaire (HLQ) among the Changsha elder population (n = 343)

Subscale/items	Obs (n = 343)	Missing [n(%)]	Median	Mean (SD)	Strongly disagree (%)	Disagree (%)	Agree (%)	Strongly agree (%)	Difficulty level (%) (95%CL)	
Feeling understood and supported by health care providers										
I have at least one healthcare provider who knows me well	343	0	3	2.66 (0.70)	4.7	33.2	53.9	8.2	37.9 (36.5–49.1)	
I have at least one healthcare provider I can discuss	343	0	3	2.68 (0.67)	5.2	27.7	60.6	6.4	32.9 (30.7–43.0)	
I have the healthcare providers I need to help me work	343	0	3	2.67 (0.69)	5.8	28.0	59.5	6.7	33.8 (31.1–43.5)	
I can rely on at least one healthcare provider	343	0	3	2.61 (0.75)	7.6	32.4	51.6	8.5	40.0 (39.2–53.2)	
				2.66 (0.70)					36.2	
2. Having sufficient information to manage my health										
I feel I have good information about health	343	0	3	2.80 (0.62)	2.9	22.4	66.8	7.9	25.3 (22.9–34.3)	
I have enough information to help me deal with my	343	0	3	2.60 (0.62)	1.7	42	50.7	5.5	43.7 (40.6–52.3))	
I am sure I have all the information I need to manage	343	0	3	2.59 (0.68)	3.8	40.5	48.4	7.3	44.3 (39.7–52.0)	
I have all the information I need to look after my health	343	0	3	2.58 (0.68)	4.1	41.1	47.8	7.0	45.2 (39.8–52.5)	
				2.64 (0.65)					39.6	
3. Actively managing my health										
I spend quite a lot of time actively managing my health	343	0	3	2.77 (0.66)	1.5	31.2	56.0	11.4	32.7 (30.2–41.0)	
I make plans for what I need to do to be healthy.	343	0	3	2.60 (0.78)	9.9	28.9	52.8	8.5	38.8 (33.5–48.0)	
Despite other things in my life, I make time to be healthy	343	0	3	2.83 (0.64)	2.3	23.6	63.0	11.1	25.9 (23.4–34.3)	
I set my own goals about health and fitness	343	0	3	2.62 (0.74)	8.2	29.2	55.4	7.3	37.4 (31.1–44.6)	
There are things that I do regularly to make myself more healthy	343	0	3	2.87 (0.63)	2.3	19.5	66.5	11.7	21.8 (18.0–28.4)	
				2.74 (0.69)					31.3	
4. Social support for health										
I can get access to several people who understand and …	343	0	3	3.03 (0.61)	0.3	16.6	63.3	19.8	16.9 (15.8–23.2)	
When I feel ill, the people around me really understand	343	0	3	2.99 (0.58)	0.9	14.6	69.1	15.5	15.5 (12.8–21.6)	
If I need help, I have plenty of people I can rely on.	343	0	3	2.94 (0.73)	3.2	20.1	56.6	20.1	23.3 (20.8–32.2)	
I have at least one person who can come to medical	343	0	3	3.15 (0.63)	1.5	9.3	62.4	26.8	10.8 (9.7–18.1)	
I have strong support from family or friends.	343	0	3	3.13 (0.62)	1.5	9.0	65.0	24.5	10.5 (8.8–16.9)	
				3.05 (0.63)					15.4	
5. Appraisal of health information										
I compare health information from different sources	343	0	3	2.73 (0.67)	2.9	30.6	56.6	9.9	33.5 (28.4–40.8)	
When I see new information about health, I check up	343	0	3	2.64 (0.69)	5.5	31.8	55.7	7.0	37.3 (32.7–45.7)	
I always compare health information from different sources	343	0	3	2.62 (0.69)	4.7	35.6	52.8	7.0	40.3 (34.9–48.0)	
I know how to find out if the health information I receive is	343	0	3	2.60 (0.69)	6.4	32.4	55.7	5.5	38.8 (34.0–47.3)	
I ask healthcare providers about the quality of the	343	0	3	2.70 (0.71)	5.0	29.7	55.4	9.9	34.7 (31.6–44.1)	
				2.66 (0.69)					36.9	
6. Ability to actively engage with healthcare providers										
Make sure that healthcare providers understand your …	343	0	4	3.39 (0.88)	2.6	12.0	35.3	43.7	6.4	49.9 (44.6–62.7)
Feel able to discuss your health concerns with a …	343	0	4	3.39 (0.92)	3.2	13.1	32.4	43.7	7.6	48.7 (43.1–61.7)
Have good discussions about your health with doctors	343	0	3	3.27 (0.90)	2.6	15.7	40.5	34.4	6.7	58.8 (53.2–72.2)
Discuss things with healthcare providers until you understand …	343	0	3	3.31 (0.89)	4.1	11.1	40.2	39.1	5.5	55.4 (49.8–68.3)
Ask healthcare providers questions to get the	343	0	4	3.47 (0.84)	1.2	11.1	35.6	44.0	8.2	47.9 (43.3–60.0)
				3.37 (0.89)						52.1
7. Navigating the healthcare system										
Find the right healthcare	343	0	4	3.34 (0.93)	2.6	19.0	25.4	47.8	5.2	47.0 (42.0–61.3)
Get to see the healthcare providers I need to	343	0	3	2.95 (1.12)	11.4	23.6	31.2	26.5	7.3	66.2 (57.5–80.7)
decide which healthcare provider you need to see	343	0	3	3.32 (0.87)	1.7	15.5	38.5	37.9	6.4	55.7 (49.1–67.0)
Make sure you find the right place to get the health …	343	0	4	3.47 (0.84)	0.9	12.5	32.9	45.8	7.9	46.3 (40.3–57.5)
Find out what healthcare services you are entitled to	343	0	3	2.88 (1.10)	12.5	20.7	36.7	25.9	4.1	69.9 (59.9–82.5)
Work out what is the best care for you	343	0	3	3.39 (0.92)	2.3	14.9	33.2	40.8	8.7	50.4 (46.1–64.0)
				3.23 (0.96)						55.9
8. Ability to find good health information										
Find information about health problems	343	0	3	3.36 (0.91)	3.8	11.4	37.6	39.9	7.3	52.8 (45.7–64.7)
Find health information from several different places	343	0	3	3.30 (0.96)	2.6	18.1	35.3	35.0	9.0	56.0 (49.0–68.5)
Get information about health so you are up to date …	343	0	3	3.08 (0.98)	5.0	23.3	37.3	28.0	6.4	65.6 (57.0–78.2)
Get health information in words you understand	343	0	4	3.50 (0.82)	0.9	11.7	31.5	49.0	7.0	44.1 (38.3–55.7)
Get health information by yourself	343	0	4	3.47 (0.76)	0.3	9.9	37.6	46.6	5.5	47.8 (41.9–57.7)
				3.34 (0.89)						53.3
9. Understanding health information well enough to know what to do										
Confidently fill medical forms in the correct way	343	0	4	3.53 (0.91)	3.2	7.6	34.1	43.4	11.7	44.9 (38.8–55.9)
Accurately follow the instructions from healthcare providers	343	0	4	3.78 (0.83)	0.6	6.7	24.2	51.3	17.2	31.5 (26.2–41.2)
Read and understand written health information	343	0	3	3.36 (0.93)	3.2	14.3	33.8	40.8	7.9	51.3 (43.6–62.7)
Read and understand all the information on medication labels	343	0	3	3.30 (0.87)	3.2	13.1	38.5	40.5	4.7	54.8 (48.2–67.1)
Understand what healthcare providers are asking you to do	343	0	4	3.76 (0.79)	0.6	5.8	24.8	54.2	14.6	31.2 (28.4–42.5)
				3.55 (0.87)						42.7

In the one-factor models, initially two scales (“2. Having sufficient information to manage own health” and “8. Ability to find good health information”) were a satisfactorily close fit without a “wiggle room.” For the other seven scales, the fit statistics were initially not satisfactory. However, after the model was modified, the model fit for all scales were perfectly fit due to the existence of correlated residuals. These correlation residuals, when contained in the model, occurred at most three times, ranging from 0.17 (scale 9) to 0.45 (scale 7). Regarding factor loadings, almost all scales had high loadings for each item at 0.45 or higher, except for one. The median loading was 0.675, which showed that almost all items were closely related to the structure of the hypothesis [37]. The one exception was in scale “7. Find out what health care services you are entitled to” (0.20).

Table 2 shows that the internal consistency of all HLQ scales was high. In addition to scale “9. Understanding health information well enough to know what to do” (composite reliability = 0.74, α = 0.77), composite reliability and Cronbach’s α ≥ 0.75 were observed in all scales. The median composite reliability was 0.78 (α = 0.78). In detail, these were: scale “2. Having sufficient information to manage own health” (composite reliability = 0.78, α = 0.78); scale “3. Actively managing own health” (composite reliability = 0.77, α = 0.78), scale “4. Social support for health” (composite reliability = 0.81, α = 0.81), scale “5. Appraisal of health information” (composite reliability = 0.79, α = 0.80); scale “6. Ability to actively engage with health care providers” (composite reliability = 0.84, α = 0.84), scale “7. Navigating the health care system” (composite reliability = 0.75, α = 0.75); scale “8. Ability to find good health information” (composite reliability = 0.78, α = 0.78), and scale “1. Feeling understood and supported by health care providers” (composite reliability = 0.85, α = 0.86), which was the highest (Table 2).

**Table 2:** Psychometric properties of Chinese-version HLQ items and scales

Item	Factor loading (95% CL)		R^2^	Composite reliability (95%CI) Cronbach’s α (*italics*)
1. Feeling understood and supported by health care providers				CR =0.85 α = 0.86
I have at least one healthcare provider who knows me well	0.68	0.55–0.77	0.46	
I have at least one healthcare provider I can discuss …	0.70	0.60–0.79	0.49	
I have the healthcare providers I need to help me work	0.79	0.72–0.86	0.63	
I can rely on at least one healthcare provider	0.87	0.81–0.93	0.76	
Fit with 1 correlated residual -χ2(ML) =0.528, p = 0.468, CFI = 1.000, TLI = 1.005, RMSEA =0.000				
2. Having sufficient information to manage my health				CR =0.78 α = 0.78
I feel I have good information about health	0.51	0.39–0.61	0.26	
I have enough information to help me deal with my …	0.76	0.68–0.84	0.58	
I am sure I have all the information I need to manage	0.72	0.60–0.83	0.52	
I have all the information I need to look after my health	0.75	0.65–0.83	0.56	
Model fit—χ2 (ML) = 5.427, p = 0.066, CFI = 0.991, TLI = 0.973, RMSEA =0.071				
3. Actively managing my health				CR =0.77 α = 0.78
I spend quite a lot of time actively managing my health	0.75	0.65–0.83	0.56	
I make plans for what I need to do to be healthy.	0.47	0.33–0.59	0.23	
Despite other things in my life, I make time to be healthy	0.81	0.72–0.88	0.65	
I set my own goals about health and fitness	0.48	0.33–0.61	0.23	
There are things that I do regularly to make myself more healthy	0.65	0.52–0.75	0.43	
Fit with 1 correlated residual—χ2(ML) = 9.566, p = 0.048, CFI = 0.989, TLI = 0.972, RMSEA =0.064				
4. Social support for health				CR =0.81 α = 0.81
I can get access to several people who understand and	0.53	0.41–0.64	0.28	
When I feel ill, the people around me really understand	0.62	0.52–0.72	0.39	
If I need help, I have plenty of people I can rely on.	0.66	0.59–0.76	0.44	
I have at least one person who can come to medical …	0.76	0.68–0.83	0.58	
I have strong support from family or friends.	0.78	0.71–0.85	0.61	
Fit with one correlated residuals - χ2 (ML) = 5.143, p = 0.2734, CFI = 0.998, TLI = 0.994, RMSEA =0.029				
5. Appraisal of health information				CR =0.79 α =0.80
I compare health information from different sources	0.63	0.52–0.71	0.39	
When I see new information about health, I check up …	0.68	0.56–0.77	0.47	
I always compare health information from different sources …	0.73	0.65–0.81	0.54	
I know how to find out if the health information I receive is	0.71	0.62–0.79	0.50	
I ask healthcare providers about the quality of the …	0.52	0.40–0.62	0.27	
Fit with one correlated residual - χ2 (ML) = 6.630, p = 0.157, CFI = 0.986, TLI = 0.994, RMSEA =0.044				
6. Ability to actively engage with healthcare providers				CR =0.84 α =0.84
Make sure that healthcare providers understand your …	0.78	0.71–0.84	0.62	
Feel able to discuss your health concerns with a …	0.83	0.76–0.89	0.69	
Have good discussions about your health with doctors	0.78	0.72–0.83	0.61	
Discuss things with healthcare providers until you understand …	0.63	0.52–0.72	0.40	
Ask healthcare providers questions to get the …	0.56	0.44–0.66	0.32	
Fit with one correlated residual - χ2(ML) = 8.928, p = 0.063, CFI = 0.981, TLI = 0.998, RMSEA =0.060				
7. Navigating the healthcare system				CR =0.75 α = 0.75
Find the right healthcare	0.59	0.50–0.67	0.35	
Get to see the healthcare providers I need to	0.52	0.41–0.63	0.28	
Decide which healthcare provider you need to see	0.78	0.69–0.85	0.61	
Make sure you find the right place to get the health …	0.71	0.59–0.81	0.51	
Find out what healthcare services you are entitled to	0.20	0.06–0.35	0.04	
Work out what is the best care for you	0.59	0.48–0.69	0.35	
Fit with three correlated residuals - χ2(ML) = 9.525, p = 0.146, CFI = 0.993, TLI = 0.983, RMSEA =0.041				
8. Ability to find good health information				CR =0.78 α = 0.78
Find information about health problems	0.67	0.55–0.75	0.44	
Find health information from several different places	0.75	0.65–0.83	0.56	
Get information about health so you are up to date …	0.64	0.54–0.71	0.40	
Get health information in words you understand	0.59	0.48–0.69	0.34	
Get health information by yourself	0.59	0.48–0.68	0.34	
Model fit—χ2(ML) = 7.502, p = 0.186, CFI = 0.994, TLI = 0.988, RMSEA =0.038				
9. Understanding health information well enough to know what to do				CR =0.74 α = 0.77
Confidently fill medical forms in the correct way	0.62	0.52–0.71	0.39	
Accurately follow the instructions from healthcare providers	0.46	0.35–0.58	0.21	
Read and understand written health information	0.76	0.64–0.856	0.57	
Read and understand all the information on medication labels	0.69	0.59–0.78	0.48	
Understand what healthcare providers are asking you to do	0.44	0.31–0.55	0.19	

Fit with two correlated residual - χ2 (ML) = 5.978, *p* = 0.113, CFI = 0.977, TLI = 0.998, RMSEA = 0.054

### Criterion validity of the Chinese HLQ scales

The correlation coefficient between the total score of the HLQ questionnaire and the validity criterion was 0.129. Table 3 shows that the correlation coefficient between the scores of nine scales and the calibration is between 0.100 and 0.191. And the correlations of the HLQ with the two composite scores that make up the SF-12 were 0.113 and 0.110. The difference was statistically significant (Table 3).

**Table 3:** Correlation analysis between the validity criterion and the score of the HLQ

Scales	correlation coefficient	*P*
Total score of the HLQ	0.129	0.011
1. Feeling understood and supported by healthcare providers	0.191	0.000
2. Having sufficient information to manage my health	0.159	0.002
3. Actively managing my health	0.113	0.026
4. Social support for health	0.122	0.016
5. Appraisal of health information	0.112	0.027
6. Ability to actively engage with healthcare providers	0.164	0.001
7. Navigating the healthcare system	0.100	0.048
8. Ability to find good health information	0.112	0.028
9. Understand health information well enough to know what to do	0.130	0.010

### Factor correlations of the nine factors

As shown in Table 4, inter-factor correlations between the nine HLQ factors ranged from 0.18 to 0.98, suggesting satisfactory discrimination, with the possible exception of scales 6, 7, 8, and 9, where the inter-factor correlations were > 0.80. For scales 2/5 = 0.90, 6/7 = 0.98, 6/8 = 0.82, 7/8 = 0.95, 7/9 = 0.84, and 8/9 = 0.89. This indicated that scales 6, 7, 8, and 9 were lacking in discriminant validity (Table 4).

**Table 4:** Factor correlations of the nine factors of the Chinese HLQ

Scale	Part I scales					Part II scales		
	1	2	3	4	5	6	7	8
2	.582							
3	.389	.793						
4	.327	.228	.318					
5	.606	.899^a^	.677	.345				
6	.379	.287	.221	.420	.373			
7	.362	.460	.353	.335	.519	.978^a^		
8	.352	.600	.507	.363	.691	.817^a^	.948^a^	
9	.176	.374	.290	.493	.443	.795	.835	.885^a^

^a^values in table indicates high factor correlations

### BSEM

To validate the previously established HLQ factor structure, a Bayesian analysis was used to fit the nine-factor model to the data. For this Bayesian analysis, the variance of the priors for the cross-loadings was set at 0.02 after several attempts, giving a 95% probability that the cross-loadings would be in the range ± 0.28. Similarly, there was also a 95% probability that the variance for the residual correlations was set at 0.02 ([35], p., 317). As a result, the model resulted in a satisfactory fit (PPP = 0.670; 95% CI for the difference between the observed and replicated Chi-square values = − 163.320, 102.750).

Table 5 shows the pattern of the statistically significant “target and non-target” factor loadings from the Bayesian analysis. The results showed that two scales (“5. Appraisal of health information” and “9. Understanding health information well enough to know what to do”) were unifactorial, with two or one statistically significant non-target loadings. Varying items of the other seven scales had statistically significant non-target loadings, indicating some multi-factorial. However, four of these scales (scale 1, 6, 7, and 8) had a pattern of strong target loadings and four statistically significant but small non-target loadings. All non-target loadings were smaller than 0.29, except for three loadings. Scale “4. Social support for health” had the strongest non-target loadings. The item P2Q13 (i.e., “Make sure you find the right place to get the health care you need”) seemed to have the most complex factor structure; it was significantly related to four factors beyond its hypothetical target construct (Table 5).

**Table 5:** Factor Loadings – Nine-factor Model of the HLQ

Item	1.Understood	2..Sufficient information	3.Active management	4.Social support	5.Appraisal	6.Active engagement	7.Navigate	8.Good information	9.Understand information
P1Q2	0.80	0.18						−0.29	
P1Q8	0.80								
P1Q17	0.78		0.20						
P1Q22	0.75							−0.14	
P1Q1		0.74							
P1Q10		0.53	0.15						
P1Q14	0.14	0.58					0.26		
P1Q23		0.60							
P1Q6		0.16	0.76						0.32
P1Q9			0.57						
P1Q13		0.14	0.63						
P1Q18			0.68						
P1Q21			0.63					0.34	
P1Q3				0.77	−0.14	−0.22			
P1Q5				0.69			−0.19		
P1Q11		0.13		0.81					
P1Q15				0.82		− 0.28			
P1Q19				0.86		−0.24			
P1Q4					0.75				
P1Q7					0.69				
P1Q12					0.62				
P1Q16					0.57				
P1Q20	0.18			0.16	0.20		−0.29		
P2Q2						0.73			
P2Q4						0.57			
P2Q7						0.62			
P2Q15						0.69			
P2Q20						0.46	0.13		
P2Q1				0.26			0.64		
P2Q8	0.15		0.25				0.42		
P2Q11							0.66		
P2Q13	−0.19			0.25		0.14	0.40	0.15	
P2Q16							0.37		
P2Q19			−0.27				0.42		
P2Q3				0.22				0.75	
P2Q6		0.31		0.14	−0.24			0.71	
P2Q10								0.47	
P2Q14			0.18					0.58	
P2Q18			−0.15					0.47	
P2Q5									0.72
P2Q9			0.19						0.48
P2Q12									0.56
P2Q17				−0.20					0.62
P2Q21									0.58

## Discussion

In this study, we measured the psychological measurement properties of the Chinese version of HLQ using older adults in Changsha as participants. The translated HLQ has a strong strength psychometric structure and original reliability. Since HLQ was not originally designed for older adults who have special characteristics, we need to understand the difficulty of the content items for older adults. In terms of the difficulty level, scale “7. Navigating the health care system” (0.56) was the most difficult among the nine scales. This is consistent with a Danish HLQ study, which found scale 7 (0.36) to have the highest difficulty level. However, the numerical value is smaller than this study’s 0.56 [11]. The difficulty level of scale “8. Ability to find good health information” was also large. The fifth item in scale “7. Find out what health care services you are entitled to” had a difficulty degree of 0.70. In today’s information society, the access to health care services and health information is limited for older adults. Low rates of online health information seeking are reported among them. Their source of information is relatively single. Studies have shown that the trend of using the internet has increased significantly, but for older adults, TV remains their most common source of health information. Overreliance on the traditional medium of television is not conducive to multi-contact and multi-source verification of information [38, 39]. A recent study that used two of the scales from the Danish HLQ showed that older adults may be more deficient in health literacy skills [40]. In short, some of the items in the Chinese version of the HLQ are a little difficult for older adults and may require adjustment.

The nine one-factor CFA models were fitted to the data for each proposed scale. Based on the criteria for factor loadings greater than 0.4 and less than 0.95, each item clearly loaded on its own factor, with only one of the 44 items loading < 0.4. (“7.5 Find out what health care services you are entitled to,” 0.20) In the meantime, the difficulty level of this item was also the largest in this questionnaire. We can guess that this item is not applicable to older adults regardless of content or difficulty level. There is also an effect on the reliability of scale 7.

The majority of the Chinese HLQ items loaded highly on their respective factors and the scales have good reliability. The fit of single-factor to the data was generally good, indicating scale homogeneity. Cronbach’s α > 0.70 is a high confidence standard [41]. In the single-factor model, all Cronbach’s α in this study were > 0.75. Regarding the composite reliability, according to Bagozzi and Yi (1989) [42], composite reliability should be greater than 0.6. Most of the composite reliability value in this study was greater than 0.80; the minimum value was 0.74. Therefore, the internal reliability of nine single-factor models was considered good. These findings are in the same range as those observed in the original psychometric studies of the English HLQ.

The Spearman correlation coefficient shown in Table 3 indicates that the HLQ has good criterion validity. Although it was lowly correlated, the reason may be that there is a lack of gold standard health literacy scale in China, so the quality of life scale affected by health literacy was selected. Despite this, SF-12 is still a recognized measurement tool and is used as a standard for analyzing the effectiveness of Chinese HLQ. Although presented as low correlation, the validity of HLQ measurements is also demonstrated to a certain extent. Scales 1–6 showed clear discriminant validity. However, the correlations of scales 2, 5, 6, 7, 8, and 9 were > 0.80, showing that the discriminant validity of these scales was not determined clearly. The high correlation of scales 7, 8, 9 has been shown in previous studies in the United Kingdom, Denmark, and Germany. A Victorian HLQ-related community application study showed that scales 6–9 has a strong correlation, which is consistent in this study [11, 28, 38, 43].

A nine-factor Bayesian model with small variance priors for both cross-loadings and residual correlations comparably fitted the data well, representing the hypothesized factor structure. This model was also used to investigate the discriminant validity of the scales. As for the statistically significant non-target factor loadings, there are three loadings > 0.29. The strongest of non-target loading is the item P1Q21 (i.e., “There are things that I do regularly to make myself healthier”) under scale "8. Ability to find good health information. The collection of information is essential before any action. It is easy to understand the association between them. The most complex factor structure was P2Q13, which is different from Victoria’s. The item P1Q16, which was the most complex factor structure in Victoria, was only significantly related to its hypothetical target structure in our results [37], which is presumably owing to cultural differences. However, it is important to note that all target loadings on these and all other scales were higher than any statistically significant non-target loadings.

From this point of view, both the original and other translated versions of the HLQ scale have powerful psychometric attributes. This robustness is related to the sensitivity of group differences related to illness of the original scale and depends on the multi-dimensional selection of the high-quality items [11]. Therefore, it can be inferred that the Chinese HLQ is a good evaluation tool of the health literacy of older adults, resulting in more in-depth and multidimensional.

### Limitations

First, compared with other studies employing CFA, the sample size in this study was relatively small because of factors related to education and age; older adults had difficulty and took a long time filling out the questionnaire. Moreover, previous studies have shown that reading skills are worse among the older population. Nevertheless, the sample size in this study is considered sufficient based on the formula for calculating sample size by scale items [43]. Second, the results of this study showed that the difficulty level of some items is high for older adults. Future research can adjust and verify the content items. Finally, although this study aimed to provide evidence to support the valid use of the HLQ among Chinese older adults, it was limited by the data provided by the participants recruited from the six districts in Changsha City, China. Future research can carry out further investigations and empirical studies on a large scale in mainland China.

## Conclusion

In China, health literacy is expected in the health system. However, there is no scale that is designed to determine the characteristics of older adults. This study showed that the Chinese version of the HLQ has strong construct and content validity and high composite reliability when applied to older adults. The nine-scale HLQ is now available to Chinese older adults, providing a more powerful multidimensional approach to assessing health literacy. The results of this study contribute to health literacy research by providing a basis for the investigation and policy formulation of health literacy for and evidence of health literacy of older adults.

## Data Availability

The data and material in this study are available from the corresponding author on reasonable request.

## Abbreviations

BSEM: Bayesian structural equation modeling
CFA: Confirmatory factor analysis
HL: Health Literacy Questionnaire
PPP: Posterior-Predictive-P value
